# Key measurement concepts and appropriate clinical outcome assessments in pediatric achondroplasia clinical trials

**DOI:** 10.1186/s13023-022-02333-6

**Published:** 2022-05-07

**Authors:** Natalie V. J. Aldhouse, Helen Kitchen, Chloe Johnson, Chris Marshall, Hannah Pegram, Sheryl Pease, Sam Collins, Christine L. Baker, Katherine Beaverson, Chandler Crews, Jill Massey, Kathleen W. Wyrwich

**Affiliations:** 1 Clinical Outcomes Assessment, Clarivate, UK; 2 Pfizer Inc, New York, USA; 3 The Chandler Project, Arkansas, USA; 4 Evelina London Children’s Healthcare, London, UK

**Keywords:** Achondroplasia, Patient-reported outcome, Clinical outcome assessment, Qualitative, Pediatric, Functioning

## Abstract

**Background:**

This study aimed to identify fit-for-purpose clinical outcome assessments (COAs) to evaluate physical function, as well as social and emotional well-being in clinical trials enrolling a pediatric population with achondroplasia. Qualitative interviews lasting up to 90 min were conducted in the US with children/adolescents with achondroplasia and/or their caregivers. Interviews utilized concept elicitation methodology to explore experiences and priorities for treatment outcomes. Cognitive debriefing methodology explored relevance and understanding of selected COAs.

**Results:**

Interviews (N = 36) were conducted with caregivers of children age 0–2 years (n = 8) and 3–7 years (n = 7) and child/caregiver dyads with children age 8–11 years (n = 15) and 12–17 years (n = 6). Children/caregivers identified pain, short stature, impacts on physical functioning, and impacts on well-being (e.g. negative attention/comments) as key bothersome aspects of achondroplasia. Caregivers considered an increase in height (n = 9/14, 64%) and an improvement in limb proportion (n = 11/14, 71%) as successful treatment outcomes. The Childhood Health Assessment Questionnaire (CHAQ) and Quality of Life in Short Stature Youth (QoLISSY-Brief) were cognitively debriefed. CHAQ items evaluating activities, reaching, and hygiene were most relevant. QoLISSY-Brief items evaluating reaching, height bother, being treated differently, and height preventing doing things others could were most relevant. The CHAQ and QoLISSY-Brief instructions, item wording, response scales/options and recall period were well understood by caregivers and adolescents age 12–17. Some children aged 8–11 had difficulty reading, understanding, or required caregiver input. Feedback informed minor amendments to the CHAQ and the addition of a 7-day recall period to the QoLISSY-Brief. These amendments were subsequently reviewed and confirmed in N = 12 interviews with caregivers of children age 0–11 (n = 9) and adolescents age 12–17 (n = 3).

**Conclusions:**

Achondroplasia impacts physical functioning and emotional/social well-being. An increase in height and improvement in limb proportion are considered to be important treatment outcomes, but children/adolescents and their caregivers expect that a successful treatment should also improve important functional outcomes such as reach. The CHAQ (adapted for achondroplasia) and QoLISSY-Brief are relevant and appropriate measures of physical function and emotional/social well-being for pediatric achondroplasia trials; patient-report is recommended for age 12–17 years and caregiver-report is recommended for age 0–11 years.

**Supplementary Information:**

The online version contains supplementary material available at 10.1186/s13023-022-02333-6.

## Background

Achondroplasia is a rare genetic condition in which bone growth is impaired. More than 250,000 people are affected worldwide [1], approximately 80% of whom were born to average-height parents [2]. Due to an autosomal dominant mutation in fibroblast growth factor receptor 3 (FGFR3) [3], people with achondroplasia have disproportional short stature; short arms with a limited range of motion, short thighs, an enlarged head, leg bowing, and hypermobile joints (hips, knees, shoulders), with the exception of the elbows, which are stiff and have a limited range of motion [2]. Individuals with achondroplasia experience disproportional growth, where growth of the trunk is not as severely affected as that of the limbs and skull [4]. Children with achondroplasia have a different schedule of developmental milestone attainment compared with children without achondroplasia [5] and may experience complications relating to impaired bone growth; craniocervical junction constriction and restrictive pulmonary disease can be of major concern during early infancy [2]. Other complications may include recurrent ear infections, sleep apnea, teeth crowding/misalignment and speech and hearing difficulties [6]. These clinical signs and symptoms impact children’s physical functioning; particularly in the context of motor skills and activities of daily living [7–15]. Living with achondroplasia can impact children’s quality of life, causing emotional and psychological impacts [7–9, 11, 14–16] and affecting aspects of social functioning (including stigma) [7, 8, 11, 14–17] and experiences at school [8, 11, 13, 15].

Treatment to date has been limited to medical and surgical interventions aimed at correcting comorbidities associated with the condition, such as an osteotomy for bowing [18], spinal decompression for foramen magnum stenosis [19], tonsillectomy and/or adenoidectomy for obstructive apnea [20], and ear tube insertion for middle ear dysfunction [21]. Uncommonly, limb-lengthening may be performed, but this is an invasive and time consuming process [22]. However, novel pharmaceutical treatments to directly address the underlying mechanism that causes the disordered bone growth in children are currently in development, including Vosoritide which is newly approved for achondroplasia [23]. To evaluate the impact of these investigational treatments on how patients feel and function, appropriate and relevant clinical outcome assessment (COA) measures are required for inclusion in clinical trials.

The US Food and Drug Administration (FDA) acknowledge that the development of new COAs is time consuming and has particular challenges in rare disease where there is an urgency to find new treatments [8]. Consequently, FDA have indicated that is it not always necessary to ‘start from scratch’ and instead, rare disease drug developers should look for ‘fit for purpose’ adjustments to endpoints for the needs of the patient group and look to measures already in use [24]. The Critical Path patient-reported outcomes (PRO) Consortium’s Rare Disease Subcommittee aim to develop fit-for-purpose endpoint measures in treatment trials for rare diseases, with the premise that existing COAs may usable or modifiable [25].

Therefore, to explore the need for a new instrument or a modifiable instrument, an initial review of qualitative literature [7–14, 16, 17, 26–28], the transcript of a 2018 FDA advisory committee meeting discussing drug development for the treatment of children with achondroplasia [15], clinical expert interviews, and existing COA measures was conducted to gain an initial understanding of the lived experience of achondroplasia and review potentially relevant COAs. Two measures, the Childhood Health Assessment Questionnaire (CHAQ) [29, 30] and Quality of Life in Short Stature Youth (QoLISSY) Brief [31] were selected for further investigation in this interview study based on their conceptual coverage, with a focus on assessment of physical functioning, pain and emotional/social well-being, respectively. Additionally, patient-reported and observer-reported outcome (ObsRO) versions of the CHAQ and QoLISSY-Brief were available which could allow some children/adolescents to self-report on their own condition. While caregiver report is necessary and appropriate for children who are too young or otherwise unable to report for themselves, patient-report for older children/adolescents should not be overlooked as the affected individual is most accurately able to report their experiences, particularly for non-observable concepts such as feelings and emotions [32].

The FDA is advancing four methodological PFDD guidance documents to address how stakeholders can collect and submit patient experience data for medical product development and regulatory decision making. These PFDD draft Guidance outline best practices to ensure that a COA is fit for its intended purpose in medical product development so that the effects seen in clinical trials can be interpreted and communicated as a clear clinical benefit that is meaningful to patients [27]. The process of selecting or developing a COA for use in a clinical development program depends on having adequately characterized the disease or condition, defined the target context of use, and conceptualized a concept of interest that represents clinical benefit [33].

This study aimed to further contribute to the conceptualization of the lived experience of the pediatric population with achondroplasia. Additionally, this study aimed to evaluate the content validity (comprehensibility, comprehensiveness, and relevance) [34] of the CHAQ and QoLISSY-Brief as measures of impact on physical functioning and well-being in children and adolescents living with achondroplasia.

## Methods

### Recruitment

This cross-sectional, non-interventional, qualitative interview study included children and adolescents living with achondroplasia and/or their caregivers. Caregivers of children aged 0–7 were interviewed alone, while children/adolescents aged 8–17 participated alongside their caregivers. Participants were identified via a specialist recruitment agency or an advocacy organization representing the community with achondroplasia, who identified participants through social media advertisements and research databases. All participants provided written informed consent/assent and ethics approval was granted by New England Independent Review Board (#20200883). Eligibility criteria are provided in Table 1. Few exclusion criteria were included, although children who had experienced limb-lengthening surgery were not invited to participate as a key objective of this study was to understand the physical and emotional impacts of the short stature and limb proportions typical of achondroplasia, and these will necessarily be different for those who have experienced limb-lengthening. Clinical characteristics, including diagnosis, were collected through parent-report as this study was conducted April–December 2020 during the height of the COVID-19 pandemic and it was not feasible for most participants to obtain clinician-reported data.

**Table 1:** Eligibility criteria

Criteria	Inclusion criteria	Exclusion
Demographics (child/adolescent living with achondroplasia)	• Child/adolescent is aged 0–17 years • (For children/adolescents participating in interview) Individual is fluent in US-English	• No restrictions
Demographics (caregiver)	• Caregiver is a primary caregiver to the child with achondroplasia, and lives with them on a full-time basis • Caregiver is aged ≥ 18 years • Caregiver is fluent in US-English	• No restrictions
Diagnosis and treatment (child/adolescent living with achondroplasia)	• Child has a clinical diagnosis of achondroplasia	• Child has a diagnosis of hypochondroplasia or any short stature condition other than achondroplasia (e.g., spondyloepiphyseal dysplasia congenital, pseudoachondroplasia, trisomy 21) • Child has any medical condition that may impact growth or where the treatment is known to impact growth, such as but not limited to hypothyroidism or hyperthyroidism, insulin-requiring diabetes mellitus, autoimmune inflammatory disease (including celiac disease, lupus nephritis, juvenile dermatomyositis, scleroderma, and others), autonomic neuropathy, or inflammatory bowel disease • Child has a history of limb lengthening surgery (defined as distraction osteogenesis, Lazaro/callostasis technique following submetaphyseal osteotomy to extend bone length)
Informed consent	• Child (where appropriate) and caregiver are willing and able to attend a 90-min (Round 1) or 60-min (Round 2) interview • Child (where appropriate) will sign assent forms to be in the study; their caregiver will sign a consent form	• Participant has any concurrent clinically significant major disease or condition that the investigator deems unsuitable for participation or other acute or chronic medical or psychiatric condition, which would also impair their ability to participate in the study

### Interview procedures

A semi-structured interview guide was developed by COA experts and was reviewed by a patient advocate and clinical expert in achondroplasia. Interviews were conducted by experienced qualitative interviewers using telephone and/or video conferencing software to allow screen sharing and video participation. Interview length ranged from 60 to 90 min; all interviews were audio-recorded and transcribed verbatim. All interviewers had Collaborative Institutional Training Initiative (CITI) Human Subjects Research certification, were trained in COA interview techniques, and attended study-specific training sessions to standardize techniques.

Interviews were conducted in two rounds to allow for adjustments to be made to the COA measures and subsequent testing. Round 1 interviews comprised combined concept elicitation and cognitive debriefing, while Round 2 interviews focused on cognitive debriefing—subsequently, a greater number of interviews were planned for Round 1 than Round 2. Sample size in Round 1 was estimated following (1) the principle of conceptual saturation to ensure that a comprehensive understanding of the participant experience was achieved [35], and (2) recommendations regarding the number of participants required to fully explore patient understand of measure items [36]. Thought leaders and industry recommendations have suggested a minimum of n = 12 interviews for both concept elicitation [35] and cognitive debriefing [36] are generally required, but the complexity and diversity of concepts should be considered and several rounds of revision/exploration may be required. As the primary purpose of the Round 2 interviews was to test any adjustments made to the COA measures as a result of the Round 1 interviews, fewer participants were required, as fewer research questions were being explored.

During concept elicitation, participants were asked open-ended questions regarding the signs, symptoms, and impacts of achondroplasia, including which aspects of achondroplasia they found most bothersome, and outcomes that they may find most meaningful following treatment.

During cognitive debriefing, participants were asked to complete the CHAQ and QoLISSY-Brief measures using ‘think-aloud’ techniques to explore relevance and understanding of each instruction, question, and response option. Caregivers of children aged 0–7, who completed the interviews alone, reviewed the ObsRO versions of the measures, while children/adolescents aged 8–11 years, who participated alongside their caregivers were encouraged to attempt to complete the PRO version; if this was too difficult, the interviewer suggested switching to the ObsRO version.

### Description of measures

#### Childhood Health Assessment Questionnaire (CHAQ)

The CHAQ is a generic measure comprising 30 items assessing the impact of illness on physical functioning in eight domains (dressing and grooming, arising, eating, walking, hygiene, reach, grip, and activities), plus two 0–100 Visual Analogue Scale (VAS) items to assess pain and global health [29, 30]. Each physical functioning item is rated on a 4-point Likert scale according to the amount of difficulty the child has experienced performing the activity in the given recall period (‘over the past week’). An additional ‘not applicable’ response is provided for activities that are not age-appropriate, and respondents are also asked to mark whether any aids, devices or assistance are required to perform each of the listed tasks. The CHAQ is recommended for use with children aged 0–19 years old; self-report (PRO completion) is recommended for children aged ≥ 8 years, while caregiver report (ObsRO completion) is recommended for children < 8 years old [29, 30]. A copy of the CHAQ is presented in the paper describing the development of the measure [29].

The wording, layout, and concepts measured in the CHAQ are consistent across the ObsRO and PRO versions of the measure, the only difference being references to ‘your child’ versus ‘you.’ Before the Round 1 interviews, references to ‘illness’ were replaced with ‘achondroplasia’ as many do not consider achondroplasia to be an illness, and to allow respondents to distinguish between achondroplasia and concurrent conditions.

#### Quality of Life in Short Stature Youth (QoLISSY-Brief)

The QoLISSY-Brief is a condition-specific measure comprising nine items (I have more trouble reaching things…, …height bothers…, …I am treated differently, …prevents me from doing things…, … I feel different…, I feel small…, I am insecure…, Because of my height I depend on others, … I get laughed at or teased [31]), with a 5-point Likert scale assessing the frequency of impact of short height on emotional/social well-being [31]. No recall period is specified. The self-report version (PRO) of the questionnaire is recommended for children aged 8–18 years old and a caregiver-report (ObsRO) version is available for children aged 4–18 years old [37]. The wording, layout, and concepts measured in the QoLISSY-Brief are consistent across the ObsRO and PRO versions of the measure, the only difference being references to ‘my child’ versus ‘me.’ The QoLISSY-Brief has not yet been published. However, a review copy of the full QoLISSY measure is available via the Pfizer Patient Reported Outcome website (https://www.pfizerpcoa.com/quality-life-short-stature-youth-qolissy).

### Analysis

Interview transcripts were reviewed in full and all identifying information (e.g., names and locations) were removed. Participants were assigned a unique ID code to allow anonymous reporting. ID codes included a recruitment number, age group, and denoted whether the speaker is the child/adolescent (P) or their caregiver (CG). For example, the ID code ‘01-[8-11]-P’ refers to a child aged 8–11 years old and ’01-[8-11]-CG’ is their caregiver.

#### Concept elicitation

The analysis took a phenomenological interpretive approach, seeking to understanding the multiple realities of participants rather than one ‘true’ reality, and focused on the perceptions, feelings and lived experiences of the participants [38]. Thematic analysis methodology (using ATLAS.ti software, version 7.5) was used to explore open-ended data [39]. Analysts familiarized themselves with the transcripts to generate a broad understanding of the reported patient experience, and then assigned descriptive codes to quotes describing specific signs, symptoms, or impacts. When multiple transcripts had been coded, analysts combined groups of related codes into domains, before comparing and contrasting those domains to assess any relationships between them.

#### Conceptual saturation

The principle of conceptual saturation was applied to confirm sufficient qualitative interviews had taken place. Conceptual saturation was assessed on the basis of concepts arising during concept elicitation. Following industry standards, the interview transcripts were grouped into four sets containing an equal number of transcripts each in the sequential order they were performed and the elicited concepts were compared between sets [33, 36]; depth of understanding of each concept was also reviewed [35]. Following completion of each set of interviews, analysts considered whether novel concepts were continuing to be described by participants, or whether depth of understanding of any concept was lacking. Interviews were concluded when these requirements were satisfied.

### Conceptual model development

A draft conceptual model of the signs, symptoms, and impacts of achondroplasia had previously been developed based on findings from a targeted review of the qualitative literature [7–14, 16, 17, 26–28], the transcript of a 2018 FDA advisory committee meeting discussing drug development for the treatment of children with achondroplasia [15], and interviews with five expert clinicians. This model was further refined to capture the concepts discussed in the qualitative interviews, and was reviewed and approved by a patient advocate.

### Concept mapping

Items from each COA measure were cross-checked against the concepts and domains included in the conceptual model to establish the conceptual coverage of the CHAQ and QoLISSY-Brief.

#### Cognitive debriefing

Data obtained via cognitive debriefing methods were subject to framework coding [40]. Analysts utilized a pre-defined code list and for each item of each measure coded how participants interacted with the COA—whether the item was found to be relevant and appropriately worded, and whether the response options and recall period were usable and understandable. The number of participants finding each item relevant and appropriate was calculated, and any difficulties experienced were explored qualitatively to determine changes that could be made to improve usability.

## Results

### Sample

A total of 48 interviews were conducted; 36 combined concept elicitation and cognitive debriefing interviews during Round 1 and a further 12 cognitive debriefing interviews in Round 2. Demographic and clinical characteristics of participants are presented in Table 2.

**Table 2:** Participant characteristics^†^

	Round 1 (N = 36)		Round 2 (N = 12)	
	Children/adolescents	Caregivers	Children/adolescents	Caregivers
**Age, mean (SD) [range]**	8 (4) [1–15]	40 (6) [26–50]	7 (6) [1–16]	39 (7) [31–50]
0–2, n (%)	8 (22)	–	3 (25)	–
3–7, n (%)	7 (19)	–	5 (42)	–
8–11, n (%)	15 (42)	–	1 (8)	–
12–17, n (%)	6 (17)	–	3 (25)	–
**Gender, n (%)**				
Male	23 (64)	7 (19)	5 (42)	1 (8)
Female	13 (36)	29 (81)	7 (58)	11 (92)
**Ethnicity**^‡^,** n (%)**				
Arabic	1 (3)	1 (3)	0	0
Asian	3 (8)	0	1 (8)	0
Black or African American	4 (11)	3 (8)	2 (17)	1 (8)
White	24 (67)	28 (78)	11 (92)	11 (92)
Hispanic	0	0	1 (8)	1 (8)
Do not wish to answer	5 (14)	5 (14)	0	0
**Caregiver relationship to child/adolescent, n (%)**				
Parent	–	36 (100)	–	12 (100)
**Highest level of education completed, n (%)**				
High school, no diploma	–	1 (3)	–	0
High school diploma	–	7 (19)	–	1 (8)
Associate's degree	–	5 (14)	–	5 (42)
Bachelor's degree	–	14 (39)	–	4 (33)
Graduate degree	–	9 (25)	–	2 (17)
**Diagnosis, n (%)**				
Before birth	16 (44)	–	6 (50)	–
At/after birth	20 (56)	–	6 (50)	–
**Treatment history, n (%)**				
Vosoritide	1 (3)	–	0	–
Surgery^§^	17 (47)	–	8 (67)	–
None	18 (50)	–	4 (33)	–
**Concomitant conditions, n (%)**				
Respiratory disease	1 (3)	–	1 (8)	–
None	35 (97)	–	11 (92)	–

*SD* standard deviation

^†^All data are caregiver-reported

^‡^Not mutually exclusive; participants could select multiple responses

^§^Including (not mutually exclusive): decompression surgeries (n = 14, 29%), adenoidectomy (n = 13, 27%), tonsillectomy (n = 11, 23%), ear tube insertion (n = 9, 19%), and others (n = 11, 23%)

### Child/adolescent experience of achondroplasia

**Fig. 1:**
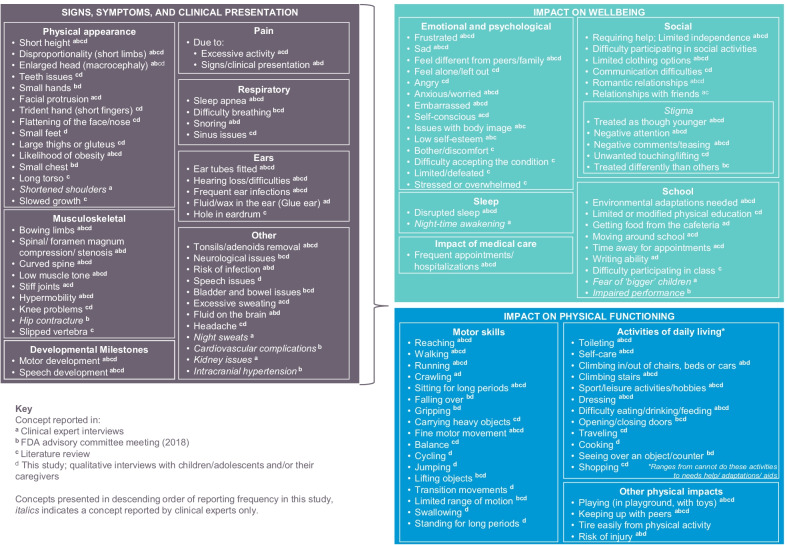
Conceptual model

A conceptual model of the lived experience of children and adolescents with achondroplasia is presented in Fig. 1. During the Round 1 interviews, pain (n = 25, 69%) was the most frequently reported symptom of achondroplasia, and difficulties reaching (n = 36, 100%), walking (n = 34, 94%), and toileting (n = 32, 89%) were commonly reported impacts on physical function. Thirty participants (83%) reported on how developmental milestones varied in comparison with children without achondroplasia, including insights into motor development and speech development. Frequency reports and example quotes are provided in Additional file 1. Twenty concepts identified in prior clinician interviews did not emerge during the child/caregiver qualitative interviews; these were mostly clinical sign/symptom concepts.

#### Most bothersome aspects of achondroplasia

Participants were asked, given all of the ways achondroplasia affects them/their child, which aspects of achondroplasia they found most bothersome. Children/caregivers named difficulty reaching, pain, short height, and disproportionality (short limbs) amongst the three most bothersome aspects of achondroplasia (Table 3). However, disproportionality was not reported among the most bothersome by adolescents aged 12–17 years, and pain and short height were not reported among the most bothersome by caregivers of children aged 0–2 years. Among these youngest children, caregivers reported difficulties with movement and health problems (e.g. frequent ear infections) as most bothersome.

**Table 3:** Top three most bothersome aspects of living with achondroplasia

Concept	No. participants naming concept amongst top 3 most bothersome aspects of achondroplasia					Example quotes
	0–2 (N = 7)	3–7 (N = 7)	8–11 (N = 12)	12–17 (N = 6)	Total (N = 32)*	
Reaching	1	2	5	3	11	– *05-[12*-*17]-P: Reaching the top shelf. […] I use a step stool, but there have been places where there have been no step stools. […] Once in a while, there’ll be a situation where I just can’t reach things, at all*
Pain	0	1	4	5	10	– *32-[8*-*11]-P: Knee pain. Sometimes when we go on a field trip and we’re doing hiking, sometimes we have to leave early because my knees are killing me*
Short height	0	3	2	3	8	– *08-[3*-*7]-CG: It’s his height, […] ‘cause he’s shorter than everyone and it makes sometimes him feel like he’s the baby*
Disproportionality (Short limbs)	3	2	2	0	7	– *07-[3*-*7]-CG: I think a big thing would be that her arms would be longer, so that she could reach and do what she wants easier*
Health issues	3	1	2	0	6	– *20-[0*-*2]-CG: He was on antibiotics all the time for ear infections*
Unable to participate; Keeping up with peers	2	1	1	1	5	– *01-[12*-*17]-P: Not being able to do sports that I really enjoy, and not being able to do them with my friends*
Negative attention/comments	0	0	4	1	5	– *31-[8*-*11]-P: Um, staring and pointing* – *39-[8*-*11]-P: When people laugh*
Physical activities	0	0	4	1	5	– *06-[8*-*11]-P: Not being able to run as fast, or walk longer distances*
Limited independence	2	1	1	0	4	*– 30-[3*-*7]-CG: The being independent, as far as, like, reaching things and that type of thing*
Motor development	3	0	0	0	3	– *29-[0*-*2]-CG: Not being able to move the way he wants to, is definitely number one*
Frequent appointments/hospitalizations	2	1	0	0	2	– *29-[0*-*2]-CG: The medical stuff, the physical stuff, it’s just been his life from the beginning. […] He just wants to be okay. He wants to be normal, and he just gets kind of upset about it, pretty frequently, actually*
Limited mobility/range of motion	0	2	0	0	2	– *14-[3*-*7]-CG: A dexterity thing. Like, being able to hold things in his arms, being able to catch balls easier, that sort of thing would be huge for him*
Speech development	1	0	0	0	1	– *20-[0*-*2]-CG: The speech delay*
Limited clothing options	0	0	0	1	1	– *04-[12*-*17]-P: Clothes is really annoying and, like, having small feet, like, now, like, especially when I was younger, and, like, only, like, the Velcro shoes were in my size and stuff, and, like, now, like, if I want a shoe, they’re not in my size, it’s definitely annoying and clothes*

*Due to interview time constraints, n = 4 participants did not complete this activity

#### Treatment outcomes

To understand meaningful treatment outcomes from the patient perspective, caregivers of children aged 0–7 years were asked about a hypothetical regular injection treatment for achondroplasia. Specifically, caregivers were asked:

- “If a treatment caused your child to grow taller in height than would be expected without the treatment, would that be meaningful? […] Would this be a successful treatment, in your opinion? […] What difference would this change make to your child’s life?”
- “If a treatment caused your child’s limbs to be more in proportion with their body than would be expected without the treatment, would that be meaningful? […] Would this be a successful treatment, in your opinion? […] What difference would this change make to your child’s life?”

Fourteen caregivers responded to these questions, and most felt that both outcomes would be meaningful. Nine caregivers (n = 9/14, 64%) considered an increase in height a successful or somewhat successful treatment outcome as this would help their child to be more independent, improve their quality of life and make the challenges faced in everyday life easier.

- *25-[3-7]-CG: I feel like it would make her be more independent in the world out here. Because, again, it’s not made for people with achondroplasia, and she wouldn’t have to depend on someone to do things for her, if she had more height.*

Eleven caregivers (n = 11/14, 71%) reported an increase in limb proportion would be considered a successful or somewhat successful treatment outcome because this would allow their child to be more independent, provide greater reach and allow independent self-care, reduce the need for aids, and improve balance.

- *20-[0-2]-CG: It would make it a little easier for him to do certain things, like get dressed, be able to use the bathroom, maybe not have to have as long as pedal extensions to drive a car in the future, or maybe not have to use stools or rely on those so much, ‘cause the outside world is not built for someone with achondroplasia.*
- *02-[0-2]-CG: You know, especially arm length, I think, would be really helpful. And well, I think it would be more meaningful than just added height overall.*

However, n = 5 caregivers felt that an increase in limb proportionality and/or an increase in height would not be meaningful treatment outcomes; some would not consider any treatment at all (n = 2/14, 14%) because they did not want to change their child, and others prioritized reduction in the risk of serious health conditions over increased height (n = 3/14, 21%) or improved limb proportionality (n = 1/14, 7%).

- *08-[3-7]-CG: No. Wouldn’t want it. Wouldn’t even use it. No. Because God made us who we are for a reason, and why change the way you are?*

#### Saturation analysis

A full understanding of each of the concepts arising in the qualitative interviews was obtained, and most (n = 94/96, 98%) of these concepts arose spontaneously within the first three sets of interviews; saturation was deemed to have been met by the end of Round 1.

### Relevance and appropriateness of the CHAQ

#### Conceptual relevance

Each CHAQ item was reported as relevant by at least n = 2 participants; the items evaluating difficulties with reach (n = 33, 92%), difficulties with activities of daily living (e.g. turn faucets on and off, n = 22, 61%; dress, n = 20, 56%; comb/brush hair, n = 19, 53%), and activities (e.g. ride bike or tricycle, n = 19, 53%; run and play, n = 16, 44%) were particularly relevant. Domain importance exploration identified that the ‘activities,’ ‘hygiene’ and ‘reach’ domains were the physical functions/limitations that children/adolescents with achondroplasia would most like a treatment to address.

The CHAQ also includes a single item global assessment of pain. A total of N = 32 participants were queried about the relevance of this item; n = 17 (53%) participants reported it to be a relevant concept for measurement. The need for assistance using aids/devices or help from another person are factored into the CHAQ scoring. A list of aids/devices is presented, alongside a write-in response box for ‘other’ items. During Round 1, 27 participants made use of the ‘other’ option noting that they needed a step stool (n = 22), light-switch extender (n = 6), and devices for toileting (n = 4).

Comparison of the items of the CHAQ against the conceptual model demonstrated good conceptual coverage of the ‘impact on physical functioning’ concepts, including the four most frequently reported impacts on motor skills and the nine most frequently reported impacts on activities of daily living, and other important concepts such as pain (see Fig. 1).

#### Understanding

##### Item wording

Many children in the age 8–11 subgroup experienced difficulties reading and/or understanding words included in the measure. One child was unable to attempt to read the measure at all, while others required caregiver input to read and/or understand specific phrases. Thus, overall, n = 15 caregivers of children aged 0–7 completed the ObsRO, n = 1 caregiver of a child aged 8–11 completed the ObsRO, n = 14 children aged 8–11 completed the PRO, and n = 6 adolescents aged 12–17 completed the PRO. Caregivers and adolescents aged 12–17 were overall able to complete the measure without difficulty, although some experienced difficulties with some sections of the measure.

Reading the measure instructions, three caregivers noted that it may be difficult to know what ‘most children at your child’s age’ are expected to do, and one caregiver reported that it may be difficult for them to think about whether their child’s limitations are due to achondroplasia. Completing the items of the measure, six caregivers misinterpreted the section heading of ‘arising’ to mean waking up in the morning, rather than, for example, standing up from a low chair or floor. Five caregivers and one adolescent were unfamiliar with one or more of the devices listed, and participants often interpreted these items to ask about any aids or devices used due to achondroplasia, rather than those specific to the activities listed. Finally, three caregivers had difficulty understanding the section heading ‘Global evaluation.’

### Response options

All caregivers were able to use the Likert scale response options as written to select an answer to each item, but four children aged 8–11 and one adolescent aged 12–17 struggled to understand the ‘not applicable’ response option. All participants were able to use the 0–100 VAS as presented to complete the pain and global evaluation items, but six felt they would be easier to interpret if the anchors were reversed and four found it difficult to choose where to place a mark along the scale.

### Recall period

Overall, most participants were able to understand the recall period and respond to all items appropriately, but two caregivers and three children aged 8–11 had difficulty understanding the phrasing “averaged over an entire day, over the past week”.

Two caregivers, three children aged 8–11 and one adolescent aged 12–17 described an incorrect recall period when asked by the interviewer, for example, the past few years (n = 2), or the past day (n = 1).

#### ObsRO: observable concepts

All caregivers could rate the majority of CHAQ items through observing their child, with most items assessing observable activities. However, six caregivers reported some difficulty in observing/rating pain severity.

#### Modifications

Based on Round 1 interview feedback, the CHAQ was amended to improve usability and understanding. These modifications form the CHAQ (adapted for achondroplasia) and are summarized in Table 4. ObsRO completion was identified as most suitable for children aged 0–11 and PRO completion by adolescents aged 12–17 only.

**Table 4:** Modifications made to the CHAQ (adapted for achondroplasia)

Element	Edit	Rationale
Instructions	Shortened and simplified wording	To make them easier to understand
Recall period	Change to ‘Over the past 7 days’	The instruction to ‘average’ difficulties experienced ‘over an entire day’ is not applicable to achondroplasia and several participants found the original description of the recall period to be confusing
‘Arising’	Replacement of ‘Arising’ with ‘Getting Up’	‘Getting up’ may better describe the applicable concepts
‘Aids and devices’ items	Capitalization of “…any of the ABOVE activities.”	To draw participants’ attention that only aids/devices used during completion of the associated activities should be considered
Aids and devices (‘hygiene,’ ‘reach,’ ‘gripping and opening things’, ‘errands and chores’)	Addition of ‘step stool’ as a listed aid/device and inclusion of an ‘other’ option	To improve relevance of the measure to achondroplasia and reduce likelihood that participants fail to acknowledge use of a step stool
‘Global evaluation’ item	Removal of ‘Global Evaluation’ heading	To make the item easier to complete
‘Pain’ and ‘global evaluation’ response scales	Replacement of 0–100 VAS with 0–10 NRS	A 0–10 NRS is easier to complete than a 0–100 VAS due to the clear numeric response options, and removes risk of inconsistencies in the length of the VAS scale due to formatting/printing of pen and paper measures
‘Pain’ response scale	Change anchors to ‘No pain’ and ‘Worst pain imaginable’	These anchors are generally considered most appropriate for pain measurement

*NRS* numeric rating scale, *VAS* visual analogue scale

In Round 2, participants cognitively debriefed the revised measure. Conceptual relevance was consistent with Round 1 results, and all items, response options and the revised instructions were well understood by all caregivers and adolescents.

### Relevance and appropriateness of the QoLISSY-Brief

#### Conceptual relevance

The QoLISSY-Brief had good conceptual coverage of impacts of achondroplasia on emotional/social well-being. The measure was reviewed by N = 35 participants, and each item was reported as relevant by at least a third of the sample (I have more trouble reaching things…, n = 34 [97%]; …height bothers…, n = 19 [54%]; …I am treated differently, n = 27 [77%]; …prevents me from doing things…, n = 31 [89%]; … I feel different…, n = 16 [48%]; I feel small…, n = 20 [57%]; I am insecure…, n = 12 [34%]; Because of my height I depend on others, n = 32 [91%]; … I get laughed at or teased, n = 23 [66%]). The nine items of the measure assess 13 of the concepts on the conceptual model (Fig. 1) (some items map to more than one concept), although this does not include the most frequently reported emotional and psychological impact ‘frustrated’ or any impacts relating to school/day care.

#### Understanding

##### Item wording

As with the CHAQ, many children in the age 8–11 subgroup experienced difficulties reading/and or understanding the QoLISSY-Brief. Two children were unable to attempt to read the measure at all, while others required caregiver input to read and/or understand specific phrases—indeed, only two children in this age group were able to complete the measure completely independently. Thus, overall, n = 15 caregivers of children aged 0–7 completed the ObsRO, n = 2 caregivers of a child aged 8–11 completed the ObsRO, n = 13 children aged 8–11 completed the PRO, and n = 5 adolescents aged 12–17 completed the PRO (one adolescent was unable to complete this section of the interview due to time constraints). However, all caregivers and three adolescents aged 12–17 interpreted and completed the measure without difficulty; two adolescents struggled to understand the meaning of ‘insecure.’

### Response options

All participants (N = 35; n = 17 caregivers; n = 18 children) were able to use the response options as written to select an answer to each item.

### Recall period

The QoLISSY-Brief lacks a defined recall period. Following debriefing, N = 27 participants were asked about the time frame they were thinking about while completing the items, and responses varied substantially (Table 5). Two participants commented that the lack of recall period made it difficult to select a response because their answer would depend on the time frame considered.

**Table 5:** Recall periods used by participants while completing the QoLISSY-Brief

Recall period used	n (%) (N = 27)	Example quote
Non-specific	6 (22)	*13-[12*-*17]-P: Recently, but also, kind of, the general picture because, again, not having much experience with the general public right now*
Right now/today	5 (21)	*20-[0*-*2]-CG: Um, I was trying to kind of think about him and his reactions and how he – his personality and how he acts today*
Past week	1 (4)	*201-[0*-*2]-CG: Just in the past, like the past week*
Past 2 weeks	2 (7)	*18-[8*-*11]-P: I was thinking about a couple of weeks ago, I think*
Past month	2 (7)	*19-[8*-*11]-P: Like, a little bit far back. […] Um, a month ago or something like that. Like, a long time ago, not, like, a week, a long time ago*
Past few months	5 (21)	*38-[8*-*11]-CG: In just the last, you know, for a while. I mean, um, say few months or something like that. In the, in the, in the present or, or near-term past. Yeah*
Past year	1 (4)	*15-[12*-*17]-P: Probably within the last year*
Past 1–2 years	3 (11)	*01-[12*-*17]-P: Mostly in the past, like, year or two*
Entire life	2 (7)	*05-[3*-*7]-CG: I was thinking about, like, his entire life here, when I answered the questions*

#### ObsRO: observable concepts

All caregivers could provide a response to all items and most found it easy to complete. However, some difficulty was reported for non-observable concepts (e.g. feel bothered, feel different, feel small, feel insecure).

#### Modifications

One change was made to the QoLISSY-Brief based on the feedback obtained during the Round 1 interviews; a recall period of 7 days was added to the measure with the instruction ‘Thinking about the past 7 days:’ prefacing the items. In addition, as with the CHAQ (adapted for achondroplasia), ObsRO completion was identified as most suitable for children aged 0–11 with PRO completion most suitable for adolescents aged 12–17 only.

In Round 2, participants cognitively debriefed the revised measure. Conceptual relevance and item understanding was consistent with the Round 1 results, and the newly added recall period was well understood by all caregivers and adolescents.

## Discussion

The findings from this interview study led to the refinement of a conceptual model of achondroplasia and an in-depth understanding of the impact of achondroplasia on individuals’ physical functioning and well-being. Discussion of the symptoms and impacts of achondroplasia found that pain, difficulties with physical function/motor skills, and difficulties with activities of daily living are of greatest importance to children and adolescents for measurement in clinical trials for achondroplasia treatments; the majority of functional impacts were discussed in-depth by the participants who provided rich insights into these experiences. When queried on factors that could be considered treatment success, caregivers of children and adolescents in this study generally confirmed height and limb proportion were important treatment outcomes, but also mentioned an expectation that treatment should address important functional outcomes such as improved reach. Our findings are in accordance with a recent review paper which observes that impacts on physical function, impacts on social function, and pain (among others), are commonly reported functional consequences of achondroplasia in natural history studies [41].

Detailed descriptions of achondroplasia signs/symptoms were obtained, although it should be noted that not all signs/clinical presentations reported in the literature and reported by clinical experts were identified in the qualitative interviews with children and their caregivers. These concepts are marked in Fig. 1 and include ‘hip contracture,’ ‘slipped vertebra,’ ‘cardiovascular complications,’ ‘kidney issues,’ and ‘intracranial hypertension.’ This is not surprising considering that many of the clinical signs/presentation in achondroplasia are heterogeneous, and are likely to be better understood by clinical experts, an important reason for including multiple sources when developing a conceptual model of disease. Similarly, many of the impacts of achondroplasia on well-being discussed in the literature, by clinical experts, and/or at the FDA advisory committee meeting did not emerge in these interviews. These concepts are also marked in Fig. 1 and include mostly emotional and psychological experiences such as ‘issues with body image,’ ‘low self-esteem,’ and difficulties with ‘relationships with friends,’ among others. However, it should be noted that emotional and psychological concepts were not probed in detail in interviews where the child was present to avoid causing undue upset, and therefore the reported frequency of these impacts may be under-represented. In addition, several participating caregivers noted during the interviews they themselves also lived with achondroplasia, and a number of the children had siblings with achondroplasia For these participants, the impact on emotional burden could be lesser than those children who were the only family member to live with achondroplasia. Nonetheless, the emotional and social well-being concerns measured in the QoLISSY-Brief were generally well-endorsed by all participants. Similarly, while all impacts on physical functioning reported in the literature also arose as concepts during this interview study, they were not reported by all participants, perhaps because some children/adolescents were living in houses that had already been adapted for their parent/caregiver’s achondroplasia (for example, bungalows with no stairs) and the impact could be lessened compared with those whose environment had not been previously adapted.

To our knowledge, this is the first study assessing the content validity of the CHAQ in a population of children with achondroplasia and/or their caregivers. The CHAQ has good conceptual coverage of the physical limitations often experienced by children/adolescents with achondroplasia, and amendments made to the original measure instructions, pain/global evaluation response scales, and ‘aids and devices’ items, which form the ‘CHAQ (adapted for achondroplasia),’ were found to produce an understandable and usable measure. Experimental data for the CHAQ will come from clinical trials. The CHAQ is currently being used in a Phase 2 study of Recifercept, a novel treatment for achondroplasia (NCT04638153), and analyses to assess psychometric validity will be conducted to confirm the remaining measurement properties of this measure in this population.

To our knowledge, this is also the first study assessing the content validity of the English version of the QoLISSY-Brief in a population of children with achondroplasia and/or their caregivers. Results suggest the QoLISSY-Brief has good conceptual coverage of the impacts on emotional and social well-being often experienced by children and adolescents with achondroplasia, with acceptable comprehensiveness and relevance. Comprehensibility appears to be adequate among adult completers of the ObsRO version of the measure and for adolescents aged ≥ 12 years completing the PRO. Our addition of a defined recall period (past 7 days) improves ease of completion and permits the monitoring of changes over time. As with the CHAQ, experimental data for the QoLISSY-Brief will come from clinical trials, and analyses collected in clinical trial NCT04638153 will inform the psychometric measurement properties of this measure for children with achondroplasia.

Based on the findings of this interview study, where the CHAQ (adapted for achondroplasia) and QoLISSY-Brief are used in a clinical trial context for the assessment of change in scores over time, ObsRO completion is recommended for children aged 0–11 with PRO completion by adolescents aged 12–17 only due to the frequency of difficulties reported by younger children. This recommendation for the age group 8–11 years differs from previous recommendations [29, 37], however the best reporter should not be based on age alone; developmental ability and the complexity of concepts measured are also relevant factors [32]. In this study several children were in a school year younger than was typical for their age.

Limitations of this study are acknowledged. This study was conducted during the height of the COVID-19 pandemic and subsequently it was not possible for most participants to easily and/or safely visit their clinicians to obtain proof of diagnosis and clinician-reported patient treatment history, or for the interviews to be conducted in person. Reported clinical demographics are therefore based on caregiver recall, but nevertheless considered to be accurate as parents were highly engaged with the diagnosis and treatment of their children. Additionally, this study was conducted in the US only and therefore findings may not be generalizable to other countries and cultures. In particular, societal attitudes to achondroplasia may differ country to country which could influence individuals’ experiences.

## Conclusions

The CHAQ (adapted for achondroplasia) and QoLISSY-Brief are relevant and appropriate measures of physical function and emotional/social well-being in pediatric achondroplasia. Patient-report versions of the CHAQ (adapted for achondroplasia) and QoLISSY-Brief are recommended for individuals with achondroplasia age 12–17 years and caregiver-report versions are recommended for age 0–11 years.

## Supplementary Information

**Additional file 1.** Overview of the concepts reported during concept elicitation (N = 36). A table of results presenting an overview of the concepts reported during concept elicitation, including the number of participants reporting experience of these concepts and an example quote from a participant describing the concept.

## Data Availability

The datasets used and/or analyzed during the current study are available from the corresponding author on reasonable request, and with permission of the study sponsor.

## Abbreviations

CHAQ: Child Health Assessment Questionnaire
CITI: Collaborative Institutional Training Initiative
COA: Clinical outcome assessment
FDA: US Food and Drug Administration
NRS: Numeric rating scale
ObsRO: Observer-reported outcome
PFDD: Patient-Focused Drug Development
PRO: Patient-reported outcome
QoLISSY: Quality of Life in Short Stature Youth
SD: Standard deviation
VAS: Visual analogue scale
