# Supplementary material for: Key measurement concepts and appropriate clinical outcome assessments in pediatric achondroplasia clinical trials
Source: Orphanet J Rare Dis. 2022 May 7;17:182. doi: 10.1186/s13023-022-02333-6 (PMC9077640; doi:10.1186/s13023-022-02333-6)
Supplement: Supplementary file 1 — Additional file 1. Overview of the concepts reported during concept elicitation (N = 36). A table of results presenting an overview of the concepts reported during concept elicitation, including the number of participants reporting experience of these concepts and an example quote from a participant describing the concept. [file 13023_2022_2333_MOESM1_ESM.docx]

Overview of the concepts reported during concept elicitation (N=36)

**Overview of the concepts reported during concept elicitation (N=36)**

| Concept | Participants reporting concept,  n (%) (N=36) | Example quote |
| --- | --- | --- |
| **Signs, symptoms, and clinical presentation** | | |
| **Physical appearance** | | |
| Shorter height | 35 (97%) | - 03-[0–2]-CG: He has a couple f-friends or, you know, friends of the family, who are about a month away from him in age and they are significantly taller than him. |
| Disproportionality | 27 (75%) | - 01-[12–17]-P: I’m shorter, like, ‘cause I’ve got this, but, like, um, I’m also, like – my limbs are disproportionate. Yeah, I think that’s the word. |
| Enlarged head (macrocephaly) | 24 (67%) | - 38-[8–11]-P: Y-yeah, but my head is little bigger. |
| Teeth issues (crowding, misalignment) | 10 (28%) | - 20-[0–2]-CG: Err, his teeth – he has a, he has a high palate, um, that the ENT told us about and his teeth seem a little bit more crowded, ‘cause his mouth is a little smaller. |
| Smaller hands | 8 (22%) | - 12-[0–2]-CG: His hands obviously are a lot smaller than most, so I mentioned, you know, gripping and, um, trying to, err, hold utensils or even cups, um, sometimes is a little bit more difficult. |
| Facial protrusion | 6 (17%) | - 02-[0–2]-CG: There’s like, frontal bossing and, um, his forehead is, you know, pretty prominent, and sticks out a little bit. |
| Trident hand | 6 (17%) | - 18-[8–11]-P: My hands are smaller, and I have, um, like, um, a real, like, space around my two middle fingers, and that, um, that other people that don’t have dwarfism have. |
| Flattening of the face/nose | 6 (17%) | - 30-[3–7]-CG: The bridge of his nose is almost, like, non-existent [laughs], I mean, it’s really, really small, the way his nose is […] that’s caused a lot of issues with, um, like, the snoring and the upper respiratory type thing, um, that’s what led to the sleep apnea. It’s, um, because of how that developed, and they said that was a lot due to the achondroplasia too. |
| Small feet | 4 (11%) | - 07-[3–7]-CG: Um, her feet are small. She’s, like, in a seven toddler shoe at this point and she’s six. |
| Large thighs or gluteus | 3 (8%) | - 35-[8–11]-CG: Okay, so, he doesn’t really have trouble getting dressed, he can put on his shirts and everything, but he does have a little bit of an issue in that his, his behind is – but, you know, little people have a, a pretty good sized backside. |
| Likelihood of obesity | 3 (8%) | - 14-[3–7]-CG: Um, well, we can see it, sadly. He is, um, technically overweight, for, for a child of his age. Um, and, and they’re calculating that based on BMI, but he – you know, he eats the same, or probably more, than an average-height child, but he’s just not able to distribute it, or, you know, run it off as much as, as another kid, so he is overweight. |
| **Musculoskeletal** | | |
| Bowing limbs (legs and arms) | 17 (47%) | - 04-[12–17]-P: Um, well, definitely when I was younger, um, with my legs being extremely bowed, like, walking long distances or riding my bike long distances, definitely hurt in those areas. |
| Spinal compression/ stenosis | 12 (33%) | - 37-[8–11]-CG: He had the compression surgery when he was, like, six-months-old, and then recently, err, they made like a window and it’s closing up and he’s got a compression in his neck and part of his spine again. |
| Curved spine | 10 (28%) | - 20-[0–2]-CG: With the back, there’s a thing that they can have called kyphosis, um, where their back kind of – their spine sticks – curves outwards, um, which tends to get better once they are more mobile. |
| Low muscle tone | 8 (22%) | - 01-[12–17]-CG: The, err, muscle, err – lack of muscle tone, but because of that, um, he, err, could not use the heavier bats, even if he, you know, even if he could find a shorter one, the heavier one would hit the ball further, so it didn’t work for him. |
| Stiff joints | 7 (19%) | - 12-[0–2]-CG: And because his elbows are more locked in place, he can’t really extend his arm out further to reach that area as well. |
| Hypermobility | 4 (11%) | - 03-[0–2]-CG: He’s definitely somewhat double-jointed. He’s very flexible and can stand up in, you know, weird ways by not bending his legs and it’s just – it’s cool, some of the things he can do. |
| Knee problems | 3 (8%) | - 41-[8–11]-P: When my arm bends, it doesn’t go straight there and that’s the same with my legs. And, like, I can’t do criss-cross applesauce or criss-cross like everyone else can, so I always have to do is go on my knees or just sit with my legs out. |
| Hip contracture | 1 (3%) | - 05-[3–7]-CG: He has got the uniquely shaped achondroplasia hips. |
| **Respiratory concepts** | | |
| Sleep apnea | 21 (58%) | - 09-[12–17]-P: Sleeping, sleeping, I have to have a mask to have air to keep going, so that I can still breathe, ‘cause without it, I stop breathing at least one a week. |
| Difficulty breathing | 19 (53%) | - 08-[3–7]-CG: The only thing that really does is his breathing, ‘cause his airwaves are narrow, so he has – he breathes a little heavy. |
| Snoring | 9 (25%) | - 30-[3–7]-CG: You know, like I said, you can hear him snore when he’s sleeping, um, but awake he doesn’t have any breathing issues. |
| Sinus issues | 4 (11%) | - 25-[3–7]-CG: She has very bad sinus issues and she had to have, um, a surgery on her throat to open it up a little bit more, because of the blockages back there, which completely closed, when it’s supposed to be open. |
| **Ear-related concepts** | | |
| Ear tubes fitted | 19 (53%) | - 201-[0-2]-CG: So, she put the ear tubes in and removed the fluid from his ears when he was a year of age and they repeated another hearing test after that, and it was normal. |
| Hearing loss/ difficulties | 17 (47%) | - 15-[12–17]-P: Yes, I do have difficulty hearing. |
| Frequent ear infections | 15 (42%) | - 20-[0–2]-CG: And then, um, we’ve had a bunch of reoccurring ear infections, which has, um, delayed his speech and we’ve had two surgeries to put tubes in his ears. |
| Fluid/wax in the ear (glue ear) | 8 (22% | - 31-[8–11]-P: […] I mean, and start, like, clogging up with wax and ever since, sometimes I have to go get them sucked out with things like the long tubes, and then I had two surgeries to get more tubes put in. |
| **Other** | | |
| Tonsil/adenoids removal | 17 (47%) | - 06-[8–11]-CG: He flew in a plane, and after that, his ears never regulated. So they removed his tonsils and adenoids. |
| Neurological issues | 12 (33%) | - 31-[8–11]-P: If I walk too long or a far distance […] it makes my legs, like, tingle a lot […] for five or ten – five minutes then – and, and then they start getting numb and I can’t walk. |
| Risk of infection | 10 (28%) | - 42-[8–11]-CG: But her respiratory, she constantly got sick, um, any kind of cold, anything really would, would put us in the hospital |
| Speech issues | 9 (25%) | - 25-[3–7]-CG: She just turned four at the end of April, and she’s just now, um, at the place where other people can understand her |
| Bladder and bowel problems | 3 (8%) | - 05-[3–7]-CG: He does have large bowel movements and he will complain that it hurts and so sometimes he stool holds, and then it’s even worse |
| Excessive sweating | 3 (8%) | - 35-[8–11]-CG: He doesn’t like the heat a lot, and, you know, kids with achondroplasia tend to get overheated easily, um, very sweaty, which he was much more sweaty as an infant, that’s a – kind of, a common thing. |
| Fluid on the brain | 3 (8%) | - 01-[12–17]-P: Hydrocephalus on my brain, and I had to get shots to get it drained, and I’ve had that ever since. |
| Headache | 3 (8%) | - 39-[8–11]-P: I get bad headaches sometimes. |
| **Developmental Milestones** | | |
| Motor development | 30 (83%) | - 15-[12–17]-CG: All of his milestones were delayed, um, but he did eventually meet them. |
| Speech development | 10 (28%) | - 29-[0–2]-CG: He doesn’t really have any, um, intellectual delays, he is – his speech is delayed, um, he has speech therapy and all that, and he wants to try to tell you something, but doesn’t know how to do it or doesn’t have the ability to because, sort of, the muscle tone and everything like that, um, has delayed it. |
| **Impact on physical functioning** | | |
| **Activities of daily living** | | |
| Toileting | 33 (92%) | - 16-[3–7]-CG: Washing her hands, she needs to step on something, or, you know, I have to pick her up to reach the sink. |
| Self-care | 26 (72%) | - 05-[3–7]-CG: He can’t reach the current shower in our room, but he can turn the shower on, like, in the tub. Like, we have a stand-up shower and he can’t reach that, but he can reach the one in the tub by himself. |
| Climbing in and out of chairs, beds or cars | 24 (67%) | - 07-[3–7]-CG: Getting in and out of cars, I mean, she just has to climb to do anything, you know, to get up on the furniture, to get up on a chair, um, where somebody else just sits down and you don’t even think, think about it. |
| Climbing stairs | 22 (61%) | - 03-[0–2]-CG: He, he goes up and down, like, I mean, he doesn’t walk up the stairs, he definitely crawls up and down the stairs, but he, he, he does that with regularity and with surprising speed now, which is terrifying. |
| Sport/ leisure activities/ hobbies | 22 (61%) | - 15-[12–17]-P: Um, like I said, basketball, err, shortest guy on the court, I can’t – it’s kind of hard for me to, like, be able to shoot over tall people, um, and other, like, gym sports, like, that require a lot of running, and I find it hard at times to go with that, hard time keeping up. |
| Dressing | 19 (53%) | - 36-[8–11]-CG: Sometimes it’s hard to get those pants right over his bottom, and he can get it up, like, he can start the process on his own, he just needs help getting it up over the butt, kind of thing. |
| Eating, drinking and feeding | 12 (33%) | - 201-[0–2]-CG: If he gets a piece of something that’s not softened enough or small enough, he would choke and cough and even vomit between his meals. |
| Opening/ closing doors | 11 (31%) | - 35-[8–11]-P: Um, well, like, if they’re tall doors, it’s hard, but if they’re small doors, I can get the handle. |
| Traveling | 7 (19%) | - 35-[8–11]-CG: He has to sit in a car seat, that’s probably not ideal, huh, [C]? So, he’s in a booster […] still because he’s little. |
| Cooking | 6 (17%) | - 09-[12–17]-P: I have to reach things, turn it on and off, or, like, when I’m cooking, like, I can’t reach none of the, like, the stuff, like, the ingredients. I have to get a stool to get to, to get, like, the ingredients for the food I’m cooking. |
| Seeing over an object/ counter | 4 (11%) | - 41-[8–11]-P: I mean, I do grow, but I don’t grow quite enough to always see. Like, I can always see a little more every time, like, I’ve been to the movies, and I can’t see all the way, ‘cause there’s either a person in front of me, that making me can’t see, or there’s, like, everything in front of me that’s shorter, but I still just can’t see because of their head or whatever else. |
| Shopping | 4 (11%) | - 13-[12–17]-P: Err, if I, like, go out in public, like to the store or to the grocery store or the mall, I usually need somebody to help me, if I’m at a strange area, or my parents, if I’m at the mall, with my friends. They’re probably my designated tall person. |
| **Motor skills** | | |
| Reaching | 36 (100%) | - 15-[12–17]-P: […] One of the only things I require help is just reaching things off cabinets or shelves, that’s the only thing I require help. |
| Walking | 29 (81%) | - 13-[12–17]-P: Mostly just, like, long walks, like, if I go to the amusement park or something, I usually am returning halfway through the trip. |
| Running | 27 (75%) | - 09-[12–17]-P: I can run fast, but for my, for my achondroplasia, but not for an average person. I’ll be, like, what, I’ll be half as slow as an – for an average person. |
| Crawling | 19 (53%) | - 03-[0–2]-CG: Err, he didn’t crawl like a typical child would, on his knees. He did more of like an army crawl, so like a full body crawl, with, like, moving his, err, arms to – and using his arms to pull him, rather, rather than using his, like, being on his knees. Um, so it was kind of like a, like a belly crawl, and then, after he did that, he did, like, a snowplough sort of thing, where his legs were fully extended when he would, sort of, crawl around, and his head would be on the ground. |
| Sitting for long periods | 15 (42%) | - 19-[8–11]-P: Sometimes, if I sit for a long time, my feet get numb or something, like, my feet and my hands get numb. |
| Falling over | 14 (39%) | - 25-[3–7]-CG: Um, in the beginning, when she was learning to walk, she would fall a lot because her – she was ready to walk, but her legs, really, were not. So she would fall a lot. Even now, if she overdoes it for the day, if she, she gets too busy dur-during the day, she’ll start falling. Um, so she has to be careful with that. |
| Gripping | 14 (39%) | - 26-[0–2]-CG: Um, holding things – um, pincher grasp she seems to have down, but like, where you would hold something with both your hands or like in one hand, she kind of has a tendency to use both hands to grip things, instead of just holding in the one hand. |
| Carrying heavy objects | 12 (33%) | - 42-[8–11]-P: Carrying a backpack with a lot of stuff in it can be a little hard […] Because there’s so much, like, pressure on my back and I can’t hold it. |
| Fine motor movements | 10 (28%) | - 42-[8–11]-CG: Um, but, like, doing her buttons or tying her shoes, um, just ‘cause of her motor skills and also, on top of her smaller fingers, she’s also a leftie, so, um, that was a little bit more difficult. |
| Balance | 9 (25%) | - 20-[0–2]-CG: Um, it took a while for him to get his balance down, for sure. Um, his, his running is, kind of, like us walking. He’s, he’s fast, but it’s very short trot. So, the, the running has been better, once he was able to gain his balance, it’s, it’s been – he still falls every once in a while, but nothing like he used to. |
| Cycling | 8 (22%) | - 36-[8–11]-CG: Yeah, he hasn’t been able to master that yet, and since his legs are, kind of, weak, he can’t push the pedals. |
| Jumping | 6 (17%) | - 14-[3–7]-CG: Jumping was hard for him at first. Um, so that was a challenge. He still can’t jump, like, on one foot, really. Um, he can’t do, like, a somersault, but we’re – we’ve been told not to let him do that anyway. |
| Lifting objects | 5 (14%) | - 13-[12–17]-P: Sometimes I can’t lift super-heavy objects, because I’m small and not very strong. |
| Transition movements | 5 (14%) | - 20-[0–2]-CG: Sitting down for him, he sits down, but he kind of plops ‘cause his arms don’t reach the floor before his butt does, whenever he goes to sit. Um, when he goes to stand, he either always has to completely bend over to push with his hands to push himself up, or he actually has mastered not using his hands at all and just standing straight up, without bending his legs, I don’t know how he does that. |
| Limited range of motion | 4 (11%) | - 13-[12–17]-P: Like in ballet class, ‘cause I have to turn my feet out to the side, it hurts my knees, because I feel like my lower legs turn out, and [inaudible – 21:42], and my knees get all twisted up and it’s not a very normal people problem. |
| Swallowing | 3 (8%) | - 201-[0–2]-CG: He has a difficulty swallowing, which was discovered when he was, err, around six months old. Err, so, since that time he has been on thickened liquids, err, and, um, kind of, different consistencies. |
| Standing for long periods | 3 (8%) | - 01-[12–17]-P: Standing up for a while also hurts my – the sides of my feet, it also hurts my back. Clearly, but yeah. Yeah, besides my feet, it also hurts my back, like, if I’m standing for a while, but other than that, I don’t – sometimes it just hurts […] if I’m, like, standing straight or, like, standing up for, like, an hour or so, but that – it normally tends to hurt then. |
| **Other physical impacts** | | |
| Playing (in the playground, with toys) | 26 (72%) | - 25-[3–7]-CG: Again, not being able to do the things that other kids do at the park. You know, not being able to get on monkey bars, or reach the swings or, you know, she still has to sit in the baby swings. |
| Keeping up with peers | 15 (42%) | - 07-[3–7]-CG: So, five kids all took off riding bikes and she was left behind, and that, that was hard to see as a parent, because it was, like, she couldn’t keep up with them. So even if she could ride a bike, she wouldn’t have been able to keep up with how fast they were going. And she just turned around and looked at me and she never said a word about it, but, but I noticed it. |
| Tire easily from physical activity | 15 (42%) | - 19-[8–11]-P: I get tired if I’m walking a long distance. |
| Risk of injury | 6 (17%) | - 07-[3–7]-CG: She loves gymnastics, and I won’t let her do it. Um, she has taught herself how to do a cartwheel, she does flips, she does head spins [laughs], um, but to put her in that setting, you know, I have – I try not to be, err, a fearing parent, but when it comes to something I think about might hurt her neck or, you know, that really – where she could really get hurt. |
| **Emotional and psychological concepts** | | |
| Frustrated | 14 (39%) | - 05-[3–7]-CG: I think sometimes he gets frustrated that he can’t keep up or that he’s not the fastest in the class. |
| Sadness | 13 (36%) | - 08-[3–7]-CG: Um, it does. Sometimes, when people are making fun of him, like, he, he’ll cry. |
| Feel different from family or peers | 5 (14%) | - 04-[12–17]-P: Everyone is just like – they look normal, you know, and, like, me, I definitely look different than other people, so it’s kind of a struggle to, like, I don’t know, realize that everyone is just a person. |
| Feel alone or left out | 4 (11%) | - 01-[12–17]-CG: So, socially, his inability to participate in some of these things does affect him. Um, and he, he, you know, has accepted that he can’t participate because it’s physically too taxing sometimes. But in our – in a small town where, you know, they’re the things kids do and play ball and play ball, then […] he, um, gets left out of a lot of things. |
| Anger | 3 (8%) | - 41-[8–11]-P: Yeah, um, I’m sometimes angry that, like, I can’t do everything that I want |
| Anxious/ worried | 2 (6%) | - 41-[8–11]-P: For surgeries I’m, like, worried-worried, but, in, like, some Doctor’s appointments, for achon or for that sort of stuff, like, being short and, like, sort of like this, and where I, like, ask him questions, what’s going to happen? But I’m not necessarily always worried, it’s sometimes just, like, I’m curious. |
| Confused | 1 (3%) | - 38-[8–11]-CG: It took her a while to understand what having achondroplasia or having dwarfism meant. And, err, yeah, it confused her a little bit. |
| Embarrassed | 1 (3%) | - 13-[12–17]-P: And, err, sometimes people throw out the M word, which I really don’t appreciate, ‘cause that’s objectifying, and not fun. And I really hate when it’s with, like, other people, like my tall people friends, because they’re embarrassed. So, they feel like they’re being made fun of too, ‘cause they’re just with me. So that’s kind of embarrassing ‘cause I don’t want to make them feel awkward, too. |
| Fearful | 1 (3%) | - 20-[0–2]-CG: He does have a stool that we’re trying to teach him how to use now, but it’s been a little difficult trying to teach him to climb and him feel – it scares him a little bit to climb up to the top of the stool. |
| Self-conscious | 1 (3%) | - 04-[12–17]-P: Oh yeah, sometimes if I’ve been with my friends, maybe I feel like self-conscious. |
| **Social impacts** | | |
| Requiring help; Limited independence | 29 (81%) | - 08-[3-7]-CG: He really wants to do it his self. He hates to ask. He hates to ask for help, but sometimes he need it, like to open up the cabinet and stuff. |
| Treated as though younger (than their actual age) | 27 (75%) | - 13-[12–17]-P: If people don’t know me, they treat me younger, because they just think small equals younger. But then when they know me, they treat me normally because they realize, I’m just as mature and I’m the same age as them, or, I’m 16, not five. |
| Difficulty participating in social activities | 13 (36%) | - 01-[12–17]-CG: He finally came up and told me that he didn’t want to go because all of the other kids at the party are ball players, more specifically baseball players, and that he felt a little out of place hanging around with a bunch of kids who play baseball. So, socially, his inability to participate in some of these things does affect him. |
| Negative attention | 11 (31%) | - 37-[8–11]-P: Sometimes people will, like, will either look at you, or point at you, and take pictures of you either […] Or they’ll try to meet you. |
| Negative comments/teasing | 10 (28%) | - 05-[3–7]-CG: I would say the worst thing is I, I don’t like it, like, when the kids on the playground were calling him Frankenstein or that he’s got to be – I would say that probably bothers me the most. |
| Limited clothing options | 8 (22%) | - 04-[12–17]-P: Things that, like, if I want to wear the same thing as my friends wear, and it’s kind of hard ‘cause it’s not going to fit me. |
| Shy around others | 2 (6%) | - 20-[0–2]-CG: He tends to be a, a lot shyer than – I have two other kids, um, but he tends to be a lot shyer around people, especially people that he doesn’t know […] So, he’s a little – he’s kind of standoffish with people that he doesn’t know. |
| Unwanted touching or lifting | 2 (6%) | - 26-[0–2]-CG: Well, maybe, because some of the neighbor kids want to pick her up and carry her and she’ll – you know, they’ll say, “Come here baby.” And she’s started saying, “I’m not a baby, I’m not a baby, a baby.” And she doesn’t like for other kids to pick her up and carry her around. She wants to get down and she wants to walk. “Don’t pick me up and I don’t need you to carry me.” |
| Communication difficulties | 1 (3%) | - 09-[12–17]-P: Oh, yeah, and I was playing outside, it just, like – it’s hard to talk to all my friends ‘cause they’re so tall, I, kind of like, have to – like, you have to – I have to move back a couple of feet to see them, like, talk to them. |
| Romantic relationships | 1 (3%) | - 13-[12–17]-P: Yeah, boys do not like me [laughs]. Boys think I’m their little sister […] [CG: In terms of being treated younger, I think that’s the answer there, is boys tend to do that and it’s difficult for her to, for example, to go to a dance and slow dance with a boy, because of the height difference.]That’s fricking awkward […] I’ve done that once, with an average-height guy [laughs]. That guy’d never want to do that again. Other than standing on chairs and things, my face is where my face does not want to be. |
| **School-related impacts** | | |
| Environmental adaptations needed | 24 (67%) | - 08-[3–7]-CG: Yes, he does have a stool at school, since a Principal assisted him with that, a, a, a stool to reach the toilet, reach the sink, and then, at his desk, he, err, has a, a pillow for his back, like, to sit up straight, ‘cause the desks are quite higher, and then a stool or something under his feet, so that he’ll be sitting up in the right position. |
| Limited or modified physical education | 16 (44%) | - 15-[12–17]-P: Gym class can be very tough, but I always find a way to fit in, and I feel like I fit in very well. So, even if there’s something I can’t do, I don’t really make a big deal out of it, but then there will be eventually something that I can do very well, so it comes, it comes and goes. |
| Getting food from the school cafeteria | 10 (28%) | - 42-[8–11]-P: Sometimes, if I can’t reach […] at lunch, either a lunch lady will help me, or one of my friends will […] they’ll just tell me and I’ll, like, I’ll just tell which one I want. It depends what food, some food I can see and some food I can’t see. |
| Moving around school | 10 (28%) | - 09-[12–17]-CG: Well, he gets five minutes extra in-between class, um, and he has use of the elevator because the school is so large, and there’s a lot of stairs. |
| Time away for appointments | 9 (25%) | - 16-[3–7]-CG: The only time she can’t attend day care, you know, if she’s sick, so you can’t send a sick kid to day care because of the other kids, so, um, just when she got sick or things like that. Um, when she had the surgery, she couldn’t go, when she was recovering, she couldn’t go, times like that. |
| Writing ability | 8 (22%) | - 25-[3–7]-CG: She’s able to hold it her way and do it her way. Not the typical way, of course, but something that’s more comfortable for her, and we do use a, a tool for her to write with. Um, and it’s like a pencil holder where you put the pencil in, and you can stick your fingers in to hold it to write. |
| **Impacts of medical care** | | |
| Frequent appointments or hospitalizations | 12 (33%) | - 201-[0–2]-CG: You know, the first year was, was very, err, eventful, it was very stressful. He had many surgeries, many hospital stays and lots of Doctor appointments and microtherapy, err, it was a very busy year. |
